# Epididymo-Orchitis in an Infant

**Published:** 1916-02

**Authors:** 


**Epididymo-Orchitis in an Infant.** A. G. Mitchell, Philadelphia, and N. J. Quinn, Atlantic City, Arch of Ped., Nov. 1915. 132 cases of gonorrhoea in infants or young children were collected from the literature but only three cases of epididymitis. Reinhard, 1914, child delivered from mother with gonorrhoea, developing urethritis at age of two weeks, epididymitis 12 days later and dying 6 days later still. Rudski, 1905, found in the literature, reports of 2 cases of epididymitis, at ages of 15 months and 12 years, respectively. The authors report a case, aged 3 months, admitted to hospital for regulation of feeding, the genitals appearing normal. 7 weeks later urethritis and epididymitis manifested themselves. Gram-negative diplococci, intra- and extra-cellular being found. The case recovered. No positive evidence as to source of infection was found, but 4 cases of urethritis followed in female infants in the same ward. (Note—The last casual statement seems to us to be the most important from the practical standpoint. Would it not be possible to introduce a routine which would absolutely prevent such an occurrence? Ed.)

